# Clinical and histological study of permanent alopecia after bone marrow transplantation*

**DOI:** 10.1590/abd1806-4841.20154013

**Published:** 2015

**Authors:** Flávia Machado Alves Basilio, Fabiane Mulinari Brenner, Betina Werner, Graziela Junges Crescente Rastelli

**Affiliations:** 1 Universidade Federal do Paraná (UFPR) - Curitiba (PR), Brazil; 2 Hospital Santa Catarina de Blumenau (HSC) - Blumenau (SC), Brazil

**Keywords:** Alopecia, Bone marrow transplantation, Drug therapy, Induction chemotherapy

## Abstract

**BACKGROUND:**

Permanent alopecia after bone marrow transplantation is rare, but more and more cases have been described, typically involving high doses of chemotherapeutic agents used in the conditioning regimen for the transplant. Busulfan, classically described in cases of irreversible alopecia, remains associated in recent cases. The pathogenesis involved in hair loss is not clear and there are few studies available. In addition to chemotherapeutic agents, another factor that has been implicated as a cause is chronic graft-versus-host disease. However, there are no histopathological criteria for defining this diagnosis yet.

**OBJECTIVE:**

the study aims to evaluate clinical and histological aspects in cases of permanent alopecia after bone marrow transplantation, identifying features of permanent alopecia induced by myeloablative chemotherapy and alopecia as a manifestation of chronic graft-versus-host disease.

**METHODS:**

data were collected from medical records of 7 patients, with description of the clinical features and review of slides and paraffin blocks of biopsies.

**RESULTS:**

Two distinct histological patterns were found: one similar to androgenetic alopecia, non-scarring pattern, and other similar to lichen planopilaris, scarring alopecia.

**CONCLUSION:**

The first pattern corroborates the literature cases of permanent alopecia induced by chemotherapeutic agents, and the second is compatible with manifestation of chronic graft-versus-host disease on scalp, that has never been described yet. The results contribute to the elucidation of the factors involved in these cases, including the development of therapeutic methods

## INTRODUCTION

Alopecia is a known adverse event of chemotherapy, usually temporary and reversible. Permanent alopecia after chemotherapy is rare, but more and more cases have been reported, associated with a great psychological and emotional disturbance.^1^ Partial hair loss cause psychological problems as intense as total loss, and this fact worsens in permanent cases.^2^

Initial reports typically involve high doses of chemotherapy (mainly busulfan and cyclophosphamide), classically used in conditioning regimens for bone marrow transplant (BMT).^2-6^ More recent reports showed cases of permanent alopecia with lower doses of chemotherapeutic agents, and related to other chemotherapy regimens, for example, in the treatment for breast adenocarcinoma with associated schedules of anthracycline and cyclophosphamide; docetaxel, carboplatin and trastuzumab; as well as paclitaxel and docetaxel alone.^2^ Busulfan, classically described in cases of irreversible alopecia, remains associated in recent cases.

The rate of permanent alopecia induced by chemotherapy is still very low and is considered a rare event. This, however, was enough for some oncology centers change their way of approaching patients, since there is a risk, even though small, to occur permanent alopecia after chemotherapy.^2,3^

The pathogenesis involved in hair loss is not yet clear. Besides chemotherapeutic agents, other factors may be involved in the cause of permanent alopecia after BMT, including chronic graft-versus-host disease (GVHD), described as a major complication of allogeneic bone marrow transplantation.^7-9^ Although there is a consensus that alopecia can be a definite clinical manifestation of chronic GVHD, there are no reports in the literature of histological findings in the scalp, and there are no well-defined criteria for diagnosis yet, in the histopathological point of view.^10^

In this study, clinical and histological characteristics of a series of 7 cases of permanent alopecia after BMT were reviewed., and these findings were correlated with etiology in each case.

### Objectives

To evaluate clinically and histologically patients with permanent alopecia after BMT. Also, to identify permanent alopecia characteristics induced by myeloablative chemotherapy and alopecia as a manifestation of chronic GVHD.

## MATERIALS AND METHODS

This is a retrospective, observational study using clinical data collected through medical records of 7 patients followed in the Dermatology and Bone Marrow Transplant Service of Hospital das Clínicas da Universidade Federal do Paraná, a national reference hospital for BMT, which performs an average of 90 transplants a year.

Patients with persistent and irreversible alopecia after 6 months of completion of chemotherapy and who underwent bone marrow transplantation were included. Children under 14 and patients over 60 years old and those with no scalp biopsy were excluded.

Patients were clinically evaluated from the dermatological point of view. The following clinical data were collected: age, sex, baseline disease before BMT, myeloablative chemotherapy regimen, type of transplantation according to the donor, rarefaction region of the scalp, hair color after BMT, hair aspects as kinking and thinning after transplantation, onset of hair loss after induction chemotherapy and time to regrowth, besides the treatments used in each case. Clinically, alopecia distribution, hair characteristics and the involvement of other hairy areas were considered. GVHD criteria were evaluated according to the "Guidelines for the diagnosis, classification, prevention and treatment of graft-versus-host disease" ^10^

Scalp biopsies slides and records filed in the Pathology Department of the hospital, of the 7 patients enrolled in the study, were reviewed. In each patient, at least two scalp biopsies were obtained. The material was collected with a 4mm punch. Specimens were fixed in 10% formalin. At least one of the samples was used in a cross-section and another one in a longitudinal section. Samples stained with hematoxylin-eosin (HE) were examined.

Biopsies were reviewed by an experienced dermatopathologist. In the longitudinal section, the evaluated criteria were: epidermis, basement membrane, type and level of the inflammatory infiltrate, presence of scarring tract, lichenoid infiltrate, apoptosis and signs of folliculitis. In the cross-section, samples were reviewed regarding the following findings: total number of follicles, amount of vellus hair and terminal follicles and terminal: vellus ratio, lichenoid infiltrate, presence of scarring tract, type and location of the inflammatory infiltrate, and signs of folliculitis.

## RESULTS

The 7 patients described underwent bone marrow transplant and developed permanent alopecia, starting immediately after induction chemotherapy and with persistence of partial alopecia after 6 months of BMT.

Most of the patients selected underwent allogeneic BMT (n=6) and one patient underwent autologous BMT (n=1). Baseline diseases that led to BMT were: severe aplastic anemia (n=2), chronic myeloid leukemia (n=2), acute myeloid leukemia (n=2) and myelodysplastic syndrome (n=1). Six patients were female and one was male. Age at time of transplantation ranged from 12-58 years, and time evolution of alopecia at the time of biopsy ranged from 1 year and 7 months to 8 years. Among other comorbidities, patient 5 had Crohn's disease and patient 6 had vitiligo.

Regarding chemotherapy regimen pre-BMT, in only one patient there was an attempt to chemotherapy before BMT (patient 3). Among the other patients, the only chemotherapy regimen to which they were subjected was myeloablative conditioning regimen immediately prior to BMT. In this standard regimen used in most pre-BMT conditioning, busulfan was used in all 7 patients (Tables 1 and 2).

**Table 1:** Clinical data related to the transplant

Patient	Gender	Baseline disease	BMT	Age at BMT
1	F	AML-M0	allogeneic	29 years
2	F	Myelodysplastic syndrome	allogeneic	35 years
3	F	AML-M3	allogeneic	50 years
4	F	Severe aplastic anemia	allogeneic	12 years
5	F	Severe aplastic anemia	allogeneic	21 years
6	F	CML	autologous	58 years
7	M	CML	allogeneic	27 years

AML= acute myeloid leukemia; CML= chronic myeloid leukemia

**Table 2:** Myeloablative chemotherapy regimen

Patient Pre-BMT conditioning chemotherapy	
1	Induction: busulfan 77 mg 6/6 h; Consolidation: ARA-C
2	Busulfan 74 mg 6/6 h- 4 days, and CPM 4470 mg - 2 days, mesna 1430 mg
3	Busulfan 53 mg 6/6 h- 4 days, CPM 3180 mg - 2 days, mesna 1g
4	Busulfan 12 mg/kg, CPM 120 mg/kg
5	Busulfan 12 mg/kg, CPM 120 mg/kg
6	Busulfan 16 mg/kg
7	Busulfan 16 mg/kg

ARA-C= cytarabine; CPM= cyclophosphamide

Among patients undergoing allogeneic BMT (n=6), only one patient showed no clinical signs of graft-versus-host disease (patient 4). The other patients had signs in various organs, involving mainly liver, lung and gastrointestinal tract. Skin was involved in 2 cases (patients 5 and 7) and it was confirmed by biopsy of cervical lesions in one of them (patient 7), presenting a perifollicular lichenoid pattern, similar to that observed in scalp (Table 3).

**Table 3:** Signs of graft-versus-host disease

Patient GVHD Criteria	
1	Chronic GVHD liver (siderosis)
2	Chronic GVHD liver - mild
3	Chronic GVHD intestine, lung and eyes
4	Absence of criteria for GVHD (heterologous, allogeneic BMT)
5	Acute GVHD skin and intestine (grade IV); Extensive chronic GVHD - skin, gastrointestinal tract, mouth, lungs, liver, vagina
6	Absence of criteria for GVHD (autologous BMT)
7	Chronic GVHD skin

GVHD = graft-versus-host disease

**Figure 1:**
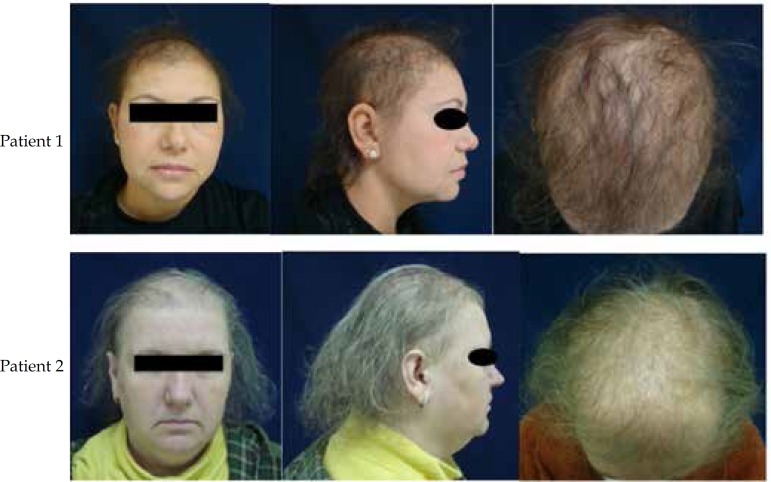
Clinical aspects – patients 1 and 2. Prominent hair thinning in frontoparietal region with fragile and sparse hair

**Figure 2:**
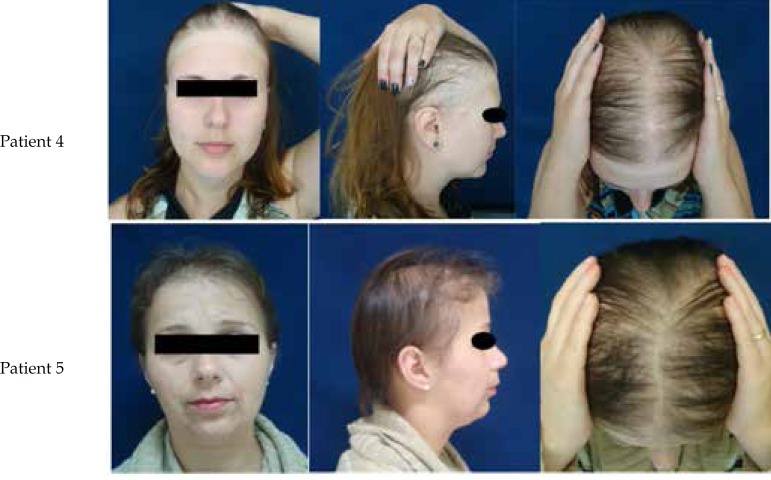
Clinical aspects – patients 4 and 5. Hair thinning in frontoparietal region, with SPARSE and fragile hair. Prominent temporal alopecia in patient 4

The beginning of hair loss occurred 1 to 2 weeks after the start of chemotherapy in all cases (anagen effluvium). The regrowth started in 8 to 12 months and remained partly, with alopecia not affecting the occipital region in most cases. This region presented the greater density compared to other areas of the scalp in all patients, while frontoparietal regions were the most affected. In one case there was also prominent alopecia in the temporal region (patient 4). The color of the hair was maintained after regrowth in most cases, except for one patient that presented a darker hair color (patient 2). There was kinking after regrowth in 4 cases and thinning of hair in all cases (Figures 1 and 2).

**Figure 3:**
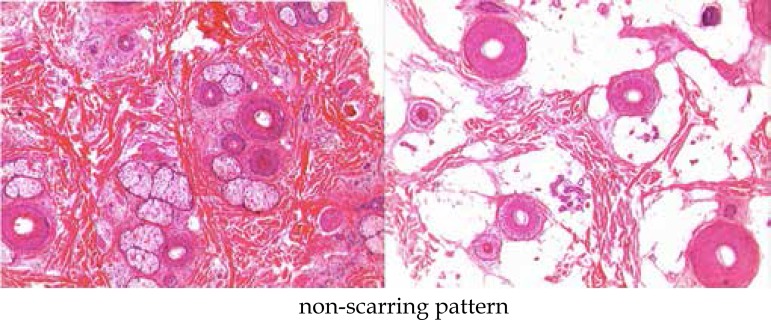
Histopathology: non-scarring pattern (HE staining). Preserved connective tissue between follicular units. Increasing in the number of vellus follicles and reduction of terminal follicles. Absence of fibrous scarring tracts. Hypodermis with terminal follicles showing variation of the diameter of the shafts

**Figure 4:**
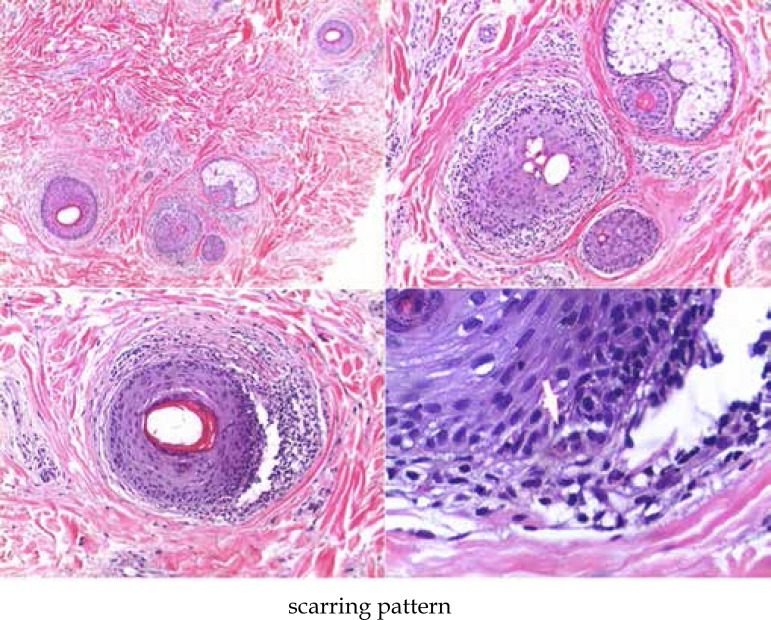
Histopathology: scarring pattern (HE staining). Marked reduction in the number of terminal follicles. Presence of concentric perifollicular fibrosis with lymphocytic infiltrate of moderate intensity. Foci of vacuolar alteration of the basal layer and rare apoptotic keratinocytes (arrow). Presence of fibrous scarring tracts

Histopathology data were evaluated in transversal and longitudinal sections. Biopsy was essential to distinguish the etiology of alopecia. In most cases a non-scarring pattern (patients 1, 3, 4, 5 and 6), with an increasing number of miniaturized follicles/ vellus hair, increase of telogens in relation to anagen and decrease of normal hair density was observed (Figure 3). Cases 2 and 7 have a pattern of scarring alopecia, with concentric fibrosis around the follicles and lichenoid inflammatory infiltrate. A classic lichenoid pattern was described in case 7. (Figure 4).

Longitudinal sections demonstrated no epidermal changes in all cases. There was vacuolar degeneration of the basal layer of the follicular epithelium in the case 7. Lymphocytic inflammatory infiltrate was seen in all cases and the location varied according to the alopecia pattern: in the cases of scarring alopecia (patients 2 and 7), the infiltrate was present in the perifollicular region. Moreover, in the other cases of permanent alopecia with non-scarring pattern, lymphocytes appeared in perivascular location, except in the case of patient 6, in which was not observed inflammatory infiltrate, despite several cuts of the sample.

Scarring tract in longitudinal section was seen in patients 2 and 7, corroborating the findings of transversal sections, and keratinocyte apoptosis was observed only in patient 7.

In table 4, we reported the total number of follicles as well as terminal: vellus ratio in each case.

**Table 4:** Follicles count and terminal: vellus ratio

Patient	N	Terminal	Vellus	Terminal:Vellus
1	19	6	13	1:2
2	30	7	21	1:3
3	14	2	12	1:6
4	25	4	19	1:5
5	17	3	14	1:4
6	16	5	11	1:2
7	12	1	11	1:11

N = total number of follicles

## DISCUSSION

From a clinical point of view, 2 types of alopecia may be induced by chemotherapy. The first is the telogen effluvium, which rarely involves more than 50% of the scalp. This occurs when a proportion of follicle, greater than normal, passes from the anagen phase to the telogen phase, and it becomes more evident in 3 to 4 months after exposure to chemotherapy. Agents that lead to telogen effluvium include: methotrexate, 5-fluorouracil, and retinoids.^2^

The second type is the anagen effluvium, and it is one of the most traumatic adverse events of chemotherapy, especially in women. Chemotherapy drugs target cells with rapid proliferation, acting in both neoplastic cells as well as in normal cells, such as cells of the matrix of hair in the anagen phase, causing a decrease of 80% of the hair.^2,5^ In the anagen effluvium, cells of the inner root sheath and the hair shaft structure suffer from the toxic effects of chemotherapy, and the final result is loss of the affected hair.^1,2^ In most cases, this type of alopecia is reversible. It begins a few days after the initiation of chemotherapy, and the regrowth occurs after the end of the medication. However, if there is telogen effluvium associated with anagen effluvium, the regrowth may be delayed and take up to 3 months. Common agents involved in the anagen effluvium are: cyclophosphamide, etoposide, topotecan and paclitaxel.^1,2^ Rare cases of this group evolve to permanent alopecia, defined as absence of hair growth or partial hair growth after a period greater than 6 months from the end of chemotherapy.^3,11^ It was first described in 1991 in six patients, after chemotherapy for conditioning for bone marrow transplantation, wherein busulfan and cyclophosphamide were used.

Busulfan has been the most implicated agent in the cases of irreversible alopecia, but other drugs, such as cyclophosphamide, thiotepa, melphalan, etoposide, carboplatin, docetaxel and paclitaxel, have also been associated with this event.^3,4,5,6,12,13^ Comparing myeloablative regimens pre-BMT, the association of busulfan with cyclophosphamide seems to be more effective than the combination of busulfan with total body irradiation in the favorable outcome of BMT.^14^ In addition, the intravenous route is more adequate than the oral, because it allows dose setting according to the pharmacokinetics, which provides fewer adverse events.^14^ It is known that the pharmacokinetics of busulfan has an individual variability, and that adjustment of dosages would be important to avoid severe adverse events. The development of permanent alopecia in a few individuals after use of busulfan may be explained by certain individual characteristics still poorly understood in pharmacokinetics of the drug. However, there is no specific dosage for preventing alopecia.^3,12,14,15^

Incidence of permanent alopecia induced by chemotherapy varies from 0.9% to 43%, and partial or complete alopecia is described in more than half of the cases of use of busulfan.^12,15^ Described risk factors are old age and prior cranial irradiation, in addition to female gender.^16^ In this study, 6 of the 7 cases occurred in female patients. However, the precise incidence and pathogenesis are unknown, and few patients are monitored by dermatologists, since the major concern is always related to hematologic diagnostics and graft viability.

Busulfan and cyclophosphamide, in conditioning regimens for bone marrow transplantation, with high-dose, were initially associated with permanent alopecia. In recent years, many other cases have been reported, most associated with chemotherapy for breast cancer, but also ovarian cancer, neuroblastoma, and malignant histiocytosis.^17^

Regarding the alopecia clinical pattern, in temporary anagen effluvium after chemotherapy, the only factor correlated to its onset was the gender of the patient, with a tendency to spare only the occipital line in men, sparing all hair implantation line in women, both occipital as frontal. Other factors, such as age or different chemotherapeutic agents, did not influence the clinical condition. The rationale for this distribution pattern is the largest amount of telogen hairs in the implantation lines. A variation in susceptibility to chemotherapeutic agents according to the scalp region, and a synergistic action with androgens, resulting in a pattern similar to androgenetic alopecia, could be involved in this distribution. It is difficult to exclude the coexistence of anagen effluvium and androgenetic alopecia, because even children can develop this pattern, which goes against hormonal influence.^1^

Histopathological evaluation of cases described in the literature is limited, restricted to 7 studies to date, and there is still no well-established pattern. Histological patterns varied. Three patients showed a reduction in the number of terminal follicles, an increasing in the number of vellus follicles, no fibrosis and absent or discrete perifollicular lymphocytic infiltration, in a pattern similar to androgenetic alopecia.^6,18^ A patient with alopecia probably caused by busulfan presented almost total loss of terminal follicles, with few vellus and no fibrosis.^5^ A case of permanent alopecia caused by docetaxel showed an uncertain meaning, with a marked reduction of anagen follicles and linear aggregates of thin epithelial structures resembling telogen germinative units.^4,19^ The latest study, published in 2011, presented 10 cases with histopathological description. Biopsies of the described group of patients (6 patients with breast cancer who used docetaxel, 3 with acute myeloid leukemia who used busulfan, and one with lung cancer who used etoposide and cisplatin) showed a non-scarring alopecia pattern, with a reduced number of terminal follicles, increased number of telogen follicles and miniaturized follicles, and presence of fiber tracts in the reticular dermis and subcutaneous, as well as Arao-Perkins bodies, which are aggregates of elastic fibers that develop deeply and underlying the follicular papila, denoting the previous location of bulbs in successively shorter folicular cycles.^20^

Permanent alopecia after chemotherapy is still little studied, but presents intriguing characteristics, because it is irreversible and non-scarring, unlike other so-called permanent alopecia. Several hypotheses have been developed to explain this finding, among them that chemotherapy could precipitate androgenetic alopecia in predisposed patients.^20^ This hypothesis would explain both clinical (predominantly thinning in androgen-dependent region of the scalp) and histopathological findings (non-scarring alopecia pattern with reduced terminal: vellus ratio). However, clinically, it is not compatible with diffuse alopecia and with short, sparse hair observed in permanent chemotherapy-induced alopecia. Furthermore, Arao-Perkins bodies are not located in the characteristic pattern described in androgenetic alopecia. Other possibilities are: reduction of stem cells in the bulge or follicular papilla after an acute injury in the matrix, apoptosis of keratinocytes and fail in restoring a new cycle with contact from the underlying dermal papilla.^20^

Several factors may be involved in the pathogenesis of permanent alopecia after BMT. In addition to the type and dose of chemotherapeutic agents, a little studied factor is the development of chronic graftversus-host disease (GVHD). In the cases described in this article, it seems to have a crucial role in the persistence of alopecia after allogeneic BMT, since in autologous BMT the GVHD does not occur.

Chronic GVHD is a multiorganic syndrome, with characteristics similar to those of autoimmune and collagen diseases, and it usually occurs 100 days after BMT, with multiple soft tissue manifestations, often mimicking well-known dermatological diseases. The most common forms of the disease are the lichenoid, sclerodermoid and vitiligoid, but phaneros changes with nail dystrophy and permanent alopecia of the scalp are also frequent.^7,8,9^

Although clinically it is a feature quite observed there are no studies on histopathological aspects of permanent alopecia in cases of chronic GVHD involving scalp. According to the "Guidelines for the diagnosis, classification, treatment and prevention of graft-versus-host disease", 2010, presence of scarring or non-scarring alopecia after chemotherapy is classified as a distinct clinical sign (observed in chronic GVHD but insufficient to establish a diagnosis by itself). A further examination is required to confirm this diagnosis and, in the case of skin or scalp, a biopsy should be performed. On the other hand, skin lesions similar to lichen planopilaris are clinically sufficient to establish diagnosis of chronic GVHD. Regarding histopathological criteria, the same guideline set specific criteria, including: combination of hypergranulosis and acanthosis with lichenoid changes and/or siringitis and/or panniculitis of eccrine units for diagnosis of chronic GVHD.^10^

In this study, we observed a scarring pattern in biopsies in 2 cases (patients 2 and 7), with lichenoid perifollicular inflammatory infiltrate, similar to what is seen in chronic GVHD's skin lesions similar to lichen planopilaris in other skin locations. Given these findings, it can be inferred that these 2 patients had a permanent alopecia condition whose etiology may be due to chronic GVHD, since both presented a distinct clinical sign (alopecia) and a specific histopathological criteria. Furthermore, both patients still showed signs of chronic GVHD in other organs (Table 3). There is not enough data in the literature to define some questions related to this topic, since not all patients develop permanent alopecia after use of busulfan or other chemotherapeutic agents. Presence of chronic GVHD could influence and may even represent a confounding factor because both conditions can coexist.

Histopathological findings of the present cases meet recent descriptions of a specific pattern of permanent alopecia induced by chemotherapy. A permanent diffuse thinning and non-scarring of the scalp seems to define the final stage of permanent alopecia induced by busulfan. In GVHD, histopathology examination with lichenoid pattern and scarring alopecia defined the condition. These 2 histologic patterns - permanent diffuse thinning and non-scarring (n=5) and lichenoid scarring/chronic GVHD (n=2) were observed in our study, featuring the 2 distinct patterns of permanent alopecia after BMT. Considering that GVHD occurs only in allogeneic BMT and the only patient in this study who underwent autologous BMT showed non-scarring pattern in the histopathological examination of the scalp, and the patient 4, the only one subjected to allogeneic BMT with no signs of GVHD, also presented this same pattern, we concluded that it would represent the effects of busulfan in the development of alopecia.

An interesting fact is that the pattern of clinical and histological condition observed in patient 4 was very similar to other cases - 1, 3, 5 and 6 - which showed signs of chronic GVHD in other organs, but with no criteria in scalp biopsy.

## CONCLUSIONS

Pathogenesis of permanent alopecia after bone marrow transplant must take into account these two main factors: chemotherapy (chemotherapy type and dose) and presence of chronic GVHD.

Two quite distinct histological patterns were found in these cases of permanent alopecia. A pattern similar to that seen in androgenetic alopecia, with a predominance of vellus/miniaturized follicles and little inflammation (5 patients), and a pattern similar to lichen planopilaris with prominent perifollicular lichenoid inflammatory infiltrate (2 patients). Although all patients studied had undergone BMT and were exposed to busulfan, busulfan is unlikely the only cause of permanent alopecia in these patients. In cases of lichenoid infiltrate the appearance is compatible with manifestation of chronic GVHD in scalp, as both presented clinical and laboratory signs of chronic GVHD in other organs, and fill clinical and histopathological criteria according to the Guidelines for the diagnosis, classification, treatment and prevention of chronic GVHD.

Histopathology proved to be a key test to diagnose alopecia as a manifestation of chronic GVHD, but much still needs to be studied on the physiopathology of permanent alopecia after BMT.

## References

[1] Yun SJ, Kim SJ (2007). Hair Loss Pattern due to Chemotherapy-Induced Anagen
Effluvium: a cross-Sectional Observation. Dermatology.

[2] Yeager CE, Olsen EA (2011). Treatment of chemotherapy-induced alopecia. Dermatol Ther.

[3] Machado M, Moreb JS, Khan SA (2007). Six cases of permanent alopecia after various conditioning
regimens commonly used in hematopoietic stem cell
transplantation. Bone Marrow Transplant.

[4] Tallon B, Blanchard E, Goldberg LJ (2010). Permanent chemotherapy-induced alopecia: Case report and
review of the literature. J Am Acad Dermatol.

[5] Tran D, Sinclair RD, Schwarer AP, Chow CW (2000). Permanent alopecia following chemotherapy and bone marrow
transplantation. Australas J Dermatol.

[6] Tosti A, Piraccini BM, Vincenzi C, Misciali C (2005). Permanent alopecia after bussulfan
chemotherapy. Br J Dermatol.

[7] Silva MM, Bouzas LFS, Filgueira AL (2005). Tegumentary manifestations of graft-versus-host disease in
bone marrow transplantation recipients. An Bras Dermatol.

[8] Oremovic L, Lugovic L, Vucic M, Buljan M, Ozanic-Bulic S (2006). Cicatricial alopecia as manifestation of different
dermatoses. Acta Dermatovenerol Croat.

[9] Tekin NS, Tekin IO, Cinar S, Altinyazar HC, Koca R, Esturk E (2005). The PUVA-turban as an alternative treatment of alopecia
associated with chronic graft versus host disease. J Am Acad Dermatol.

[10] Bouzas LF, Silva MM, Tavares RCBS, Moreira MCR, Correa MEP, Funke VAM (2010). Diretrizes para o diagnóstico, classificação, profilaxia e
tratamento da doença enxerto contra hospedeiro crônica. Rev Bras Hematol Hemoter.

[11] Baker BW, Wilson CL, Davis AL, Spearing RL, Hart DN, Heaton DC (1991). Busulphan/cyclophosphamide conditioning for bone marrow
transplantation may lead to failure of hair regrowth. Bone Marrow Transplant.

[12] Ljungman P, Hassan M, Békássy AN, Ringdén O, Oberg G (1995). Busulfan concentration in relation to permanent alopecia in
recipients of bone marrow transplants. Bone Marrow Transplant.

[13] de Jonge ME, Mathôt RA, Dalesio O, Huitema AD, Rodenhuis S, Beijnen JH (2002). Relationship between irreversible alopecia and exposure to
cyclophosphamide, thiotepa and carboplatin (CTC) in high-dose
chemotherapy. Bone Marrow Transplant.

[14] Bredeson C, LeRademacher J, Kato K, Dipersio JF, Agura E, Devine SM (2013). Prospective cohort study comparing intravenous busulfan to
total body irradiation in hematopoietic cell
transplantation. Blood.

[15] Perez-Crespo M, Betlloch I, Ballester I, Lucas A, Mataix J, Niveiro M (2009). Irreversible alopecia due to busulphan in a 7-year-old
girl. Eur J Dermatol.

[16] Vowels M, Chan LL, Giri N, Russell S, Lam-Po-Tang R (1993). Factors affecting hair regrowth after bone marrow
transplantation. Bone Marrow Transplant.

[17] Palamaras I, Misciali C, Vincenzi C, Robles WS, Tosti A (2011). Permanent chemotherapy-induced alopecia: a
review. J Am Acad Dermatol.

[18] Prevezas C, Matard B, Pinquier L, Reygagne P (2009). Irreversible and severe alopecia following docetaxel or
paclitaxel cytotoxic therapy for breast cancer. Br J Dermatol.

[19] Tallon B, Blanchard E, Goldberg LJ (2013). Permanent chemotherapy-induced alopecia: histopathologic
criteria still to be defined. 2013. J Am Acad Dermatol.

[20] Miteva M, Misciali C, Fanti PA, Vincenzi C, Romanelli P, Tosti A (2011). Permanent alopecia after systemic chemotherapy: a
clinicopathological study of 10 cases. Am J Dermatopathol.

